# Isoproterenol-Induced Permeability Transition Pore-Related Dysfunction of Heart Mitochondria Is Attenuated by Astaxanthin

**DOI:** 10.3390/biomedicines8100437

**Published:** 2020-10-20

**Authors:** Roman Krestinin, Yulia Baburina, Irina Odinokova, Alexey Kruglov, Irina Fadeeva, Alena Zvyagina, Linda Sotnikova, Olga Krestinina

**Affiliations:** Institute of Theoretical and Experimental Biophysics, Russian Academy of Sciences, 142290 Pushchino, Moscow Region, Russia; rkrestinin@bk.ru (R.K.); byul@rambler.ru (Y.B.); odinokova@rambler.ru (I.O.); krugalex@rambler.ru (A.K.); aurin.fad@gmail.com (I.F.); leavi@inbox.ru (A.Z.); linda_sotnikova@mail.ru (L.S.)

**Keywords:** rat heart mitochondria (RHM), mitochondrial permeability transition pore (mPTP), regulatory proteins, reactive oxygen species (ROS), cardiolipin (CL)

## Abstract

Mitochondria are key organelles of the cell because their main function is the capture of energy-rich substrates from the cytoplasm and oxidative cleavage with the generation of carbon dioxide and water, processes that are coupled with the synthesis of ATP. Mitochondria are subject to oxidative stress through the formation of the mitochondrial permeability transition pore (mPTP). Various antioxidants are used to reduce damage caused by oxidative stress and to improve the protection of the antioxidant system. Astaxanthin (AST) is considered to be a dietary antioxidant, which is able to reduce oxidative stress and enhance the antioxidant defense system. In the present investigation, the effect of AST on the functional state of rat heart mitochondria impaired by isoproterenol (ISO) under mPTP functioning was examined. It was found that AST raised mitochondrial respiration, the Ca^2+^ retention capacity (CRC), and the rate of TPP^+^ influx in rat heart mitochondria (RHM) isolated from ISO-injected rats. However, the level of reactive oxygen species (ROS) production increased. In addition, the concentrations of cardiolipin (CL), Mn-SOD2, and the proteins regulating mPTP rose after the injection of ISO in RHM pretreated with AST. Based on the data obtained, we suggest that AST has a protective effect in rat heart mitochondria.

## 1. Introduction

Mitochondria are the center of cellular energy production and a major source of reactive oxygen species (ROS). Mitochondria produce superoxide anions as byproducts of electron leakage from mitochondrial respiratory chain complexes I and III [1]. Maintenance of the structural and functional integrity of mitochondria is the most important precondition for normal cellular function, because mitochondria play a key role in energy metabolism, as well as in sustaining the cellular redox state and regulating apoptosis. Since mitochondria are the main source of reactive oxygen species (ROS), their dysfunction results in oxidative stress, which causes the cells to enter into a diseased state [2]. Oxidative stress is considered to be one of the risk factors for the development of cardiovascular diseases [3]. It is the main cause of various human disorders such as metabolic syndrome and neurodegenerative, cardiovascular, and inflammatory diseases, as well as age-related failures. Mitochondrial dysfunction may provoke the development of oxidative-stress-associated diseases [4,5]. The impairment of mitochondrial function is responsible for various human diseases, including cardiovascular pathologies [6].

Moreover, oxidative stress causes the mitochondrial membrane to become permeable, initiating the formation of the mitochondrial permeability transition pore (mPTP) [7]. The opening of the mPTP induced by Ca^2+^ is accompanied by changes in the conformation of the regulatory proteins of the mPTP and the release of pro-apoptotic factors (AIF, cytochrome *c*, etc.) [8]. In addition, if the mPTP opens, its high conductivity can cause the swelling of mitochondria due to the osmotic pressure of the solutes of the matrix, rupture of the outer mitochondrial membrane (OMM), a decrease in the potential of the mitochondrial membrane (ΔΨm), and the depletion of cellular ATP, which leads to apoptosis or necrosis [9,10]. The protein composition of the mPTP has not been determined, and proteins that were earlier believed to be the structural components of the mPTP are now considered now to be its regulators [7]. Among these pore-regulating proteins are the voltage-dependent anion channel (VDAC) in the OMM [7], adenine nucleotide translocase (ANT) in the inner mitochondrial membrane (IMM) [11], subunit *c* (sector Fo of ATP synthase) [12], and phosphodiesterase of cyclic nucleotide (CNPase) localized in the OMM and mitoplasts [13,14]. 

The main mechanism of toxicity of free radicals is the peroxidation of membrane lipid components, which leads to the impairment of membrane function. This function can be performed by cardiolipin (CL), which is a phospholipid localized inside the IMM, which is especially rich in unsaturated fatty acids [15]. Thus, mitochondrial CL is a possible target for the actions of ROS, either because of its high content of unsaturated fatty acids or its location in the IMM near the site of ROS formation, mainly at the level of Complex I [16] and complex III [17] of the mitochondrial respiratory chain. CL plays an important role in mitochondrial bioenergetics, stimulating the activity of key proteins of the IMM, namely, several anion carriers and some complexes of the electron transport chain (ETC) [18] and is the main phospholipid involved in maintaining mitochondrial function and myocardial health [19]. A loss of CL in heart disorders enhances ROS production and strengthens cardiolipin peroxidation, which leads to mitochondrial dysfunction and, ultimately, cardiomyocyte death [20].

It is known that cardiac function is regulated by various antioxidant defense mechanisms; however, in heart disorders, antioxidant defenses are impaired, and an increase in ROS production suppresses the capacity for antioxidant protection [21,22]. Dietary antioxidants can reduce oxidative stress [23,24], increase the protection of the mitochondrial antioxidant system [25] and, as a result, prevent the development of cardiovascular diseases. Among these antioxidants are carotenoids, which are divided into carotenes and xanthophils. The group of carotenes involves β-carotene and lycopene, and the group of xanthophylls contains lutein, canthaxanthin, zeaxanthin, violaxanthin, capsorubin, and astaxanthin [26,27]. Of greatest interest for research is astaxanthin (AST), because it is obtained from natural sources in the form of an ester of fatty acids or as a conjugate of proteins in food products [2]. AST is found in many living organisms, mainly from marine surroundings. It is contained in different concentrations in unicellular microalgae, plankton, krill, and other seafood such as salmon, trout, and crustaceans, including crayfish and shrimp [28]. AST reduces oxidative stress and protects cells, such as HeLa and undifferentiated PC12 rat pheochromocytoma cells, from it. In addition, AST maintains a high mitochondrial membrane potential and stimulates respiratory activity [29]. There is great interest around AST due to its biochemical characteristics, mainly as a potent antioxidant, a role in which it is approximately ten times more effective than β-carotene or lutein and about 100 times more effective than α-tocopherol [30,31]

Recently, we showed that the addition of AST to rat heart mitochondria (RHM) is capable of improving the functional state of RHM and increasing the respiratory control index (RCI) and P/O ratio [32]. Moreover, oral administration of AST under oxidative stress induced by isoproterenol (ISO) increases the concentration of subunits of the respiratory chain complexes and ATP synthase both in intact RHM and after direct addition of AST to mitochondria. In addition, we observed that the pretreatment of rats with AST increased the activity of respiratory chain complexes and ATP synthase in RHM injured by ISO and suggested that AST prevents oxidative damage by increasing the efficiency of the mitochondria [33]. 

The aim of this study is to investigate the effect of AST on the impairment of RHM function induced by ISO under mPTP opening. Changes occurring under these conditions in left ventricle (LV) tissue, ROS production, the amount of CL, the levels of regulatory proteins, and superoxide dismutase were examined. 

## 2. Experimental Section

### 2.1. Animals and Treatment

In experimenters, we used Wistar male rats (16 animals, weight 240–250 g, and age two months). All animals were kept under the same conditions in a room with a temperature of 22 °C and fed a standard diet with access to water and food. The rats were divided into four groups (four rats in each group); therefore, four independent repetitions were made for each group. The rats in group 1 were the control, rats in group 2 were administrated AST, rats in group 3 were injected with isoproterenol (ISO, isoprenaline hydrochloride, Sigma 15627, Saint-Louis, Missouri, USA), and rats in group 4 were administered AST and then two weeks later, injected with ISO. Therefore, rat groups 1 and 3 were used as controls, and the animals in groups 2 and 4 were administrated AST (Natural, Hong Kong, China) for four weeks (hereafter referred to as AST groups 2 and 4). AST (5%, 150 mg/kg) [32,33] was dissolved in olive oil and administered orally using plastic feeding tubes (15 ga × 78 mm, Instech, Plymouth Meeting, Plymouth, PA, USA). Control animals received an equal amount of olive oil as a vehicle. Experiments were carried out as described in [34,35] with some modifications. Two weeks later, rats from groups 3 and 4 were injected with ISO dissolved in saline (100 mg/kg body weight) twice with an interval of 24 h to induce mitochondrial disturbances, and the rats from groups 1 and 2 were injected with saline. ISO was injected subcutaneously to animals. After a second injection of ISO or saline, 14 days later, fragments of the LV were cut off for histological analysis, and RHM were isolated from the remaining hearts of rats in each group. The experiments were carried out according to the Regulations for Studies with Experimental Animals (Decree of the Russian Ministry of Health of 12 August 1997, No. 755). The protocol was approved by the Commission on Biological Safety and Ethics at the Institute of Theoretical and Experimental Biophysics, Russian Academy of Sciences (March 2019, protocol N18/2019).

### 2.2. Histological Analysis

To get a true picture of fibrotic heart injury, samples from identical regions of the left ventricle (LV) of the rat heart (the upper right quadrant above the left coronary artery) were taken for the analysis. For the histological analysis, fragments of the LV were cut off with a scalpel as quickly as possible from the whole heart immediately after withdrawal from the chest and rapidly washed in cold phosphate-buffered saline to remove blood [36]. Then, LVs were fixed in neutral buffered formalin (NBF) for 24 h at room temperature according to the standard technique [36]. After the termination of fixation, LV fragments were washed three times from excessive phosphates in distilled water and immersed in medium O.C.T. Compound Tissue Tek (Sakura, Tokio, Japan) for no less than 12 h at +4 °C. Sets of three successive 9 μm thick cross-sections of LV were prepared using a Shandon CRYOTOME 620E (Thermo Fisher Sci., Waltham, MA, USA) with steps of 30 μm. Each set of three neighboring sections was stained with hematoxylin and eosin (H&E) and with two differential trichrome staining methods. To obtain a general picture of heart failure, histotopograms were taken by gluing on a Nikon Eclipse Ti-E microscope station (Nikon, Tokio, Japan) and using Nis Elements software AR4.13.05 (Build933). To determine the type of fibrosis, subendocardial (mixed type of section of longitudinal and transverse muscle bundles of the myocardium) and subepicardial (predominantly cross-sections of myocardial bundles) regions of the myocardium were estimated separately and compared.

Total fibrosis of the myocardium was assessed by two techniques: Masson’s trichrome staining and Lillie’s trichrome staining [37]. The percentage of fibrous myocardial changes was estimated from digitized images (no less than five regions of analysis from each section) using the noncommercial software ImageJ (https://imagej.nih.gov/ij/). Maximal fibrosis observed on a section (blue regions) was calculated as the area occupied by the connective tissue plus cardiac myocytes × 100, as described previously [38]. To exclude artefacts, the regions of intramural vessels stained in blue, as well as perivascular collagen and endocardial collagen, were not considered. The increment in the degree of fibrosis in experimental groups was estimated as the difference in the degree of maximal fibrosis of sections expressed as a percentage of blue regions of control samples from group 1. The data are given as the mean ± standard deviation.

### 2.3. Isolation of Rat Heart Mitochondria

Mitochondria were isolated from the whole heart according to the method described in [35]. The heart was freed from blood vessels, minced and homogenized with the help of a medium volume glass homogenizer containing 75 mM sucrose (Sigma S7903, Saint-Louis, MO, USA), 10 mM Tris-HCl (pH 7.4), 225 mM mannitol (Sigma M4125, Saint-Louis, MO, USA), 0.5 mM EDTA (Sigma E9884, Saint-Louis, MO, USA), 0.5 mM EGTA (Sigma E3889, Saint-Louis, MO, USA), and 0.1% BSA (Sigma A6003, Saint-Louis, MO, USA). After that, the homogenate was centrifuged at 1000× *g* for 10 min, and the pellet was deleted. Mitochondria in the supernatant were precipitated at 8500× *g* for 10 min. The mitochondrial pellet was washed and suspended in isolation medium without EDTA or BSA (8500× *g*, 10 min). All procedures were conducted at 4 °C. The protein content in mitochondria was determined using the Bradford assay. The concentration of protein in an RHM suspension remained 30–35 mg/mL. 

### 2.4. Evaluation of the Mitochondrial Function

The medium for incubating mitochondria (1 mg protein/mL) contained 125 mM KCl, 10 mM Tris (pH 7.4), and 2 mM K_2_HPO_4_ at a temperature of 25 °C. As respiratory substrates, we used glutamate (5 mM) and malate (5 mM). The respiratory activity rates were determined after the addition of 150 µM ADP (V_st.3_) and 1.5 µM oligomycin (Sigma 75351, Saint-Louis, Missouri, USA) (V_st.4_) to RHM in a closed chamber. The respiratory control index (RCI) was calculated as the ratio of V_st.3_ to V_st.4_. The oxygen consumption rates (V_st.2_, V_st.3_, and V_st.4_; ng-atom O min^−1^ mg^−1^ of protein) were estimated. 

Changes in the rate of TPP^+^ (Tetraphenylphosphonium ion) influx (V^TPP+^_in_) and the Ca^2+^ retention capacity (CRC) of RHM were measured in a multifunctional chamber (1-mL) with integral TPP^+^- and Ca^2+^-sensitive electrodes (Nico, Moscow, Russia), and a Clark-type O_2_ electrode. The Clark-type O_2_ electrode was used to measure the oxygen consumption rate [39]. The mPTP opening in RHM was induced by a threshold [Ca^2+^] concentration (each addition of Ca^2+^ contained 50 nmol per mg of protein). The threshold calcium concentration [Ca^2+^] is the concentration of Ca^2+^ added to a mitochondrial suspension at which Ca^2+^ ions (when accumulating in mitochondria) induce mPTP opening. The Ca^2+^-induced dissipation of the membrane potential was defined as the TPP^+^ influx rate (V^TPP+^_in_, nmol min^−1^ mg^−1^ of protein), because a change in this indicator reflects a drop in membrane potential. RHM swelling was determined by measuring changes in light scattering in a mitochondrial suspension at 540 nm (A540) on a Tecan I-Control Infinite 200 spectrophotometer at 25 °C. The standard incubation medium for the swelling assay contained 125 mM KCl, 10 mM Tris, 2 mM KH_2_PO_4_, 5 mM glutamate, and 5 mM malate. The concentration of mitochondrial proteins in a well was 0.5 mg protein/mL. Swelling was initiated by the addition of 260 nmol of Ca^2+^ per mg of protein. The swelling process was characterized by the time needed to reach the half-maximal light scattering signal (T_1/2_).

### 2.5. Measurement of ROS in Rat Heart Mitochondria

The concentration of hydrogen peroxide in a mitochondrial suspension was measured as described previously [35] in a standard medium supplemented with 5 mM glutamate, 5 mM malate, 40 µM Amplex Red (Thermo Fisher Scientific A12222, Waltham, MA, USA), horseradish peroxidase (HRP, Sigma P8250, Saint-Louis, MO, USA) (3 U/mL), and 10 µM EGTA. Where indicated, the medium also contained 200 µM Ca^2+^, 25 µM menadione (Sigma M5625, Saint-Louis, MO, USA) + 1 mM EGTA, and 10 µg/mL antimycin A (Sigma A8674, Saint-Louis, MO, USA) + 1 mM EGTA. Resorufin accumulation was traced using a plate fluorimeter (Infinite 200 Tecan, Mannedorf, Switzeland) in 96-well plates at excitation and emission wavelengths of 530 and 595 nm. The maximal rate of hydrogen peroxide production was calculated from the sharpest slope of the curves. For the quantitative assessment of hydrogen peroxide, fluorescence was calibrated as the excess concentration of hydrogen peroxide at the end of the measurement period. In order to avoid light-induced resorufin formation, the fluorescence was measured once or twice per minute.

The rate of superoxide anion (SA) production was assessed using the highly sensitive chemiluminescent probe 3.7-dihydro-2-methyl-6-(4-methoxyphenyl)imidazo[1,2-a]pyrazine-3-one (MCLA, Sigma 87787, Saint-Louis, MO, USA) [40]. The kinetics of MCLA-derived chemiluminescence (MDCL) was recorded using a plate reader (Infinite 200 Tecan) in accordance with an earlier described protocol [41]. The incubation medium contained the same additions as those used for hydrogen peroxide measurements, except that Amplex Red and HRP were replaced by 20 µM MCLA and some samples contained superoxide dismutase (100 U/mL, Sigma S7571, Saint-Louis, MO, USA) to calibrate the luminescent response. Ca^2+^ and ROS can induce mitochondrial damage and mPTP opening when the activation of ROS production is followed by fast inhibition due to the loss of important cofactors [42]. Therefore, we determined the average MDCL from the beginning of measurements to the achievement of maximal MDCL.

### 2.6. Measurement of the Content of Cardiolipin in Heart Mitochondria

The level of CL in heart mitochondria was assessed using the CL-selective fluorescent probe nonyl-acridine orange (NAO, Sigma A7847, Saint-Louis, MO, USA) [43]. Since the most efficient quenching of the NAO fluorescence by CL occurs at an NAO/CL ratio (mol/mol) of 2:1, we calibrated the fluorescent signal of 10 µM of NAO by increasing the quantity of mitochondria with steps of 10–20 µg protein/mL. For calibration, NAO, mitochondrial suspensions of different volumes, and standard incubation medium without respiratory substrates were added to wells of a 96-well plate (total volume of the mixture 100 µL/well). After 20 min incubation at 37 °C with mixing for 3 s two times per min, the fluorescence (480/525 nm for excitation/emission, respectively) was recorded using a plate reader (Infinite 200 Tecan, Mannedorf, Switzeland). We assumed that the minimal levels of fluorescence corresponded to quantities of mitochondria that contained 5 nmol of CL.

### 2.7. Preparation of Samples, Electrophoresis, and Immunoblotting of Mitochondrial Proteins

To prepare samples to determine the concentrations of OxPhos Complexes and mitochondrial proteins and aliquots of a mitochondrial suspension (2 mg/mL) were taken from the chamber (Materials and Methods section “*Evaluation of mitochondrial function*”), placed in an Eppendorf tube, and solubilized in Laemmli buffer. Samples used for determining the concentration of OxPhos Complexes were heated to 37 °C for 3 min, while samples used for the detection of alterations in the expression of mitochondrial proteins were heated to 95 °C for 3 min and applied to the gel. Changes in the levels of ETC enzymes were detected with a Total Oxphos Rodent WB Antibody Cocktail (ab 110413, monoclonal antibodies, Cambridge, UK). The Oxphos Antibody Cocktail consisted of Complex V (CV) alpha subunit (ATP5A-55 kDa), Complex III (CIII) core protein 2 (Cytochrome b-c1 complex subunit 2, UQCRC2-48 kDa), Complex IV (CIV) subunit I (mitochondrially encoded cytochrome *c* oxidase I, MTCO1-40 kDa), Complex II (CII) subunit (Succinate dehydrogenase [ubiquinone] iron–sulfur subunit, SDHB-30 kDa), and Complex I (CI) subunit NDUFB8-20 kDa (NADH dehydrogenase (ubiquinone) 1 beta subcomplex subunit 8, NDUFB8). The polyclonal anti-ANT antibody (1:1000), the polyclonal VDAC antibody (1:1000), the monoclonal anti-ATPG1/G2/G3 antibody (subunit *c*) (1:1000), anti-ATPF1 (subunit *b*) (1:1000), and the polyclonal anti-SOD-2 (1:5000) antibody were obtained from Abcam (Cambridge, UK); the monoclonal anti-CNPase antibody was obtained as described previously [44] and used at a dilution of 1:10,000. The Tom20 antibody (1:1000 dilution; Cell Signaling, Danvers, MA, USA) was used as a loading control. The immunoreactivity was detected using an appropriate secondary antibody conjugated to horseradish peroxidase (Jackson Immuno Research, West Grove, PA, USA). Peroxidase activity was detected with ECL (Bio-Rad, Hercules, CA, USA) using the ChemiDoc Touch Imaging System (Bio-Rad, Hercules, CA, USA). Protein bands were quantified by densitometry (Image Lab program, Bio-Rad, Hercules, CA, USA).

### 2.8. Statistical Analysis

For statistical analysis, relative levels of protein density were expressed as the mean ± SD of at least four independent experiments. For statistical analysis, we used one-way ANOVA and a proper post-hoc analysis (Student–Newman–Keuls) (Supplementary Materials). We compared each parameter with the corresponding control only. This study design favors an ANOVA type 2 analysis. Student–Newman–Keuls (SNK) tests are recommended for determining the differences among several experimental groups. The SNK test is used for all pairwise comparisons of mean responses among different treatment groups to declare an observed difference as being statistically significant. Differences were considered significant at *p* < 0.05.

## 3. Results

### 3.1. Histological Analysis of Cryosections of the Left Ventricle of the Rat Heart after Administration of AST and Injection of ISO

**Figure 1:**
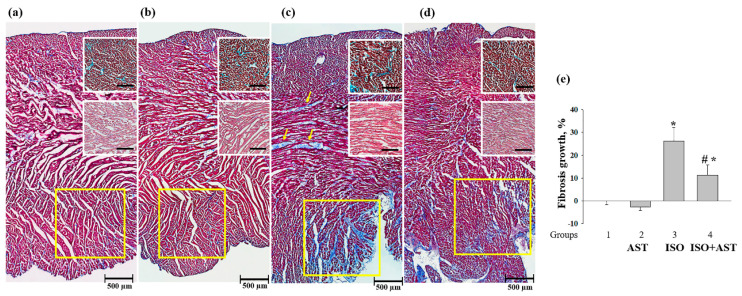
Histotopograms (columns) of left ventricle (LV) tissue from the hearts of rats. (**a**) Control, group 1; (**b**) rats exposed to chronic astaxanthin (AST) treatment, group 2; (**c**) rats injected with isoproterenol (ISO), yellow arrows show fibrous myocardial damage, group 3; (**d**) rats exposed to chronic AST treatment and injected with ISO, group 4. Light microscopy; the main images—Masson’s trichrome staining (collagen/fibrosis is stained with blue, muscle and other tissues are stained with red, and cell nuclei are stained with brown); upper insets—magnified fragments of the subendocardial zone of the myocardium with predominantly transverse sections of myocardial fibers; Lillie’s trichrome staining (collagen/fibrosis is shown in blue; muscle and other tissues are in red-brown; cell nuclei are in brown-black); middle insets—magnified fragments of the median zone of the myocardium; H&E (cell nuclei are in blue, erythrocytes are in red, muscle tissue is in pink); lower fragments contoured in yellow are the most typical regions of the subendocardial zone of the myocardium; a part of the basic histogram; (**e**) a diagram reflecting fibrosis growth in the tissue of the left ventricle from each group of rats. Data are presented as means ± SDs of five independent experiments. * *p* < 0.05 indicates a significant difference in the protein level relative to the control (group 1). # *p* < 0.05 compared to RHM isolated from ISO-injected rats (group 3). The statistical significance of the differences between the pairs of the mean values was evaluated using an ANOVA type 2 (Student–Newman–Keuls) test.

To assess the degree and nature of myocardial damage and determine its localization, survey transmural histotopograms of samples of the left heart ventricles of animals from all groups were examined. In addition, injuries in the subepicardial (Figure 1, upper insets), median (Figure 1, middle insets), and subendocardial myocardial (Figure 1, lower insets) zones were estimated and compared. The nature of fibrosis and the degree of hypertrophy were revealed by comparing the zones with predominantly transverse (Figure 1, upper insets) and longitudinal sections of muscle fibers of the myocardium (Figure 1, middle and lower insets).

A comparison of the data obtained for groups 1 and 2 did not reveal any differences in any of the myocardium zones (Figure 1a,b). All histological characteristics of these groups corresponded to the standards of the myocardial structure and architectonics of this age group of Wistar rats. In the heart fragments of rats in groups 3 and 4, subendocardial injuries of the myocardium were predominant (Figure 1c,d). In samples of rats from group 4, fibrous myocardial damage was more localized and less pronounced (Figure 1d). The subepicardial and median zones of the cardiac wall were almost not affected. This differed from samples from group 3 in which signs of fibrous alterations in the median myocardial zone and regions of fusion of swollen muscle fibers and the appearance of subsegmentary contractures were observed. The increment of fibrous alterations in group 3, as estimated using macros from ImageJ, was significantly greater than that in LV samples from group 4 (mean ± SD 26.25 ± 4.3% vs. 11.35 ± 2.75%, *p* < 0.05; *n* = 10). A comparative examination of histotopograms showed that, in both group 3 and group 4, there were signs of myocardial hypertrophy; however, in samples from group 4, this process was less pronounced and was restricted predominantly by the subendocardial zone and partially by the median zone, as distinct from samples of group 3, where this process was of the general type (compare Figure 1c,d with Figure 1a).

### 3.2. Effect of AST Administration and ISO Injection on Respiratory Activity in Rat Heart Mitochondria

At the next step, we measured the respiratory activity and RCI in RHM isolated from rats of each group. The oxygen consumption rates in different states and RCI values are given in Table 1.

It can be seen that there were no substantial differences in the rate of oxygen consumption in states 2 and 3 (V_st.2_ and V_st.3_) in RHM from group 2 compared with RHM from group 1 (RHM from group 2 vs. group 1). On the contrary, the oxygen consumption rates (V_st.2_ and V_st.3_) in RHM of rats injected with ISO decreased by 36% and 58%, respectively, compared with RHM from group 1 (RHM from group 3 vs. group 1). After pretreatment with AST in combination with ISO, the oxygen consumption rates (V_st.2_ and V_st.3_) in RHM increased by 40% and 2.3 times, respectively, in comparison with those of the RHM of group 3 (RHM from group 4 vs. group 3). The oxygen consumption rate (V_st.4_) in RHM isolated from group 3 decreased by 9% (RHM from group 3 vs. group 1).

There was no substantial difference in the RCI in RHM from group 2 compared with that of the RHM from group 1 (RHM from group 2 vs. group 1). On the contrary, the RCI in the RHM of rats injected with ISO decreased by two times compared with the RHM from group 1 (RHM from group 3 vs. group 1). Pretreatment with AST in combination with ISO increased the RCI by two times relative to the RCI of group 3 (RHM from group 4 vs. group 3). The administration of AST improved the functional state of the RHM.

### 3.3. Effects of AST and ISO on the Level of Enzymes in the Electron Transport Chain in Rat Heart Mitochondria under mPTP Opening

**Figure 2:**
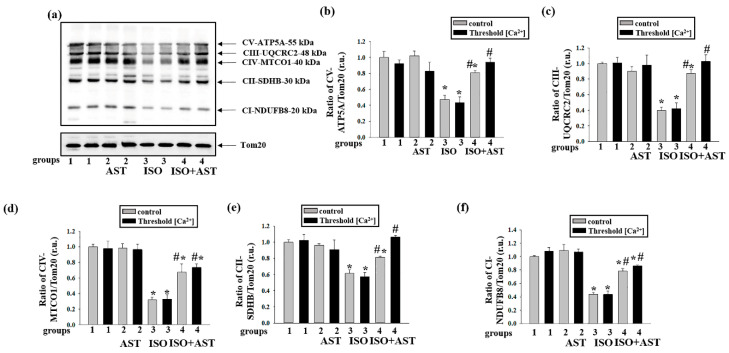
Effects of the administration of AST and injection of ISO on the concentrations of mitochondrial respiratory chain complexes in rat heart mitochondria under mitochondrial permeability transition pore (mPTP) opening. Protein samples were extracted from control and threshold [Ca^2+^] samples and subjected to Western blotting. Changes in mitochondrial complexes were detected using a Total OXPHOS Rodent WB Antibody Cocktail. The immunodetection of Tom20 was used as a loading control. (**a**) Immunostaining with the OXPHOS antibody cocktail and Tom20; (**b**–**f**) quantification of immunostaining by computer-assisted densitometry (subunit α of complex V (CV-ATP5A-55 kDa), cytochrome b–c1 complex subunit 2 of complex III (CIII-UQCRC2-48 kDa), mitochondrially encoded cytochrome c oxidase I subunit of complex IV (CIV-MTCO1-40 kDa), succinate dehydrogenase [ubiquinone] iron–sulfur subunit of complex II (CII-SDHB-30 kDa), and dehydrogenase [ubiquinone] 1 β subcomplex subunit 8 of complex I (CI-NDUFB820 kDa), respectively). The data are presented as the means ± SDs of three independent experiments. * *p* < 0.05 indicates a significant difference in the protein level relative to the control (group 1). # *p* < 0.05 compared with RHM isolated from ISO-injected rats (group 3). The statistical significance of the differences between the pairs of mean values was evaluated using an ANOVA type 2 (Student–Newman–Keuls) test.

**Figure 3:**
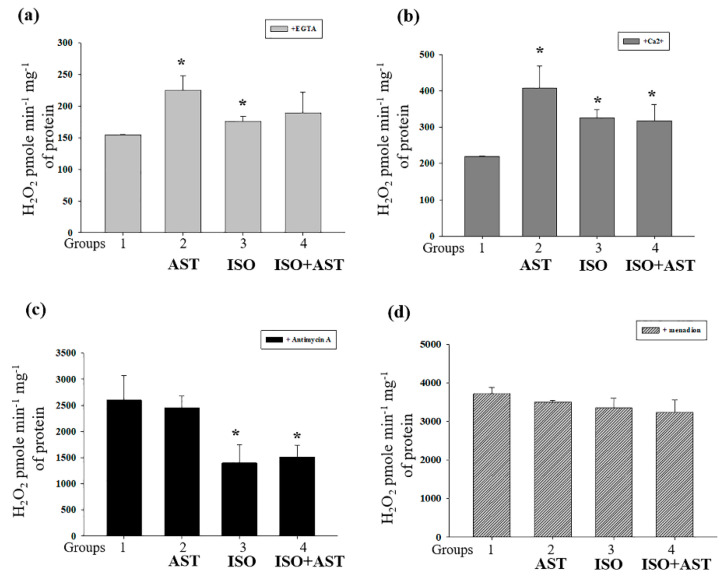
Effect of the administration of AST and injection of ISO on H_2_O_2_ production in rat heart mitochondria. (**a**) H_2_O_2_ production in the presence of EGTA; (**b**) H_2_O_2_ production in the presence of threshold [Ca^2+^]; (**c**) H_2_O_2_ production in the presence of Antimycin A; (**d**) H_2_O_2_ production in the presence of menadion. The data are presented as means ± SDs of four independent experiments. * *p* < 0.05 indicates a significant difference in the protein level relative to the control (group 1). The statistical significance of the differences between the pairs of the mean values was evaluated using the ANOVA type 2 (Student–Newman–Keuls) test.

Earlier, we showed that AST enhances the activity of the respiratory chain complexes and increases the concentration of basic subunits of these complexes in intact RHM [33]. Here, we examined changes in the concentrations of the main subunits of the respiratory chain complexes in RHM isolated from each group of rats upon the opening/closing of the mPTP (Figure 2). Figure 2a shows a Western blot stained with Oxphos antibodies for the basic subunits of ETC complexes. Tom20 was used as a loading control. Figure 2b–f demonstrate the data on immunostaining obtained by computer-assisted densitometry and gives the ratios of the levels of proteins to Tom20. We observed that the injection of ISO decreased the levels of the CV, CIII, CIV, CII, and CI subunits by approximately 50%, 60%, 70%, 40%, and 45%, respectively, when mPTP was closed compared with the control (RHM from group 3 vs. group 1, grey columns). Under these conditions, but when the mPTP was open, the contents of these subunits diminished by 40%, 60%, 70%, 45%, and 65%, respectively (RHM from group 3 vs. group 1, Figure 3b–f, black columns). The combined actions of AST and ISO led to increases in the concentrations of the CV and CII subunits by 30% and 25%, respectively, when the mPTP was closed, relative to when the ISO was applied alone (RHM from group 4 vs. group 3, grey columns; Figure 2b,e). The concentrations of the CIII, CIV, and CI subunits were upregulated by two times under these conditions (RHM from group 4 vs. group 3, grey columns; Figure 2b–d,f). When the mPTP was open, the concentrations of the CII and CI subunits increased by two times compared to when ISO was applied alone (RHM from group 4 vs. group 3, black columns, Figure 2e,f), and the concentrations of the CV, CIII, and CIV subunits increased by more than two times (RHM from group 4 vs. group 3, black columns, Figure 2b–d). AST abolished the effect induced by ISO.

### 3.4. Effects of AST and ISO on H_2_O_2_ Production in Rat Heart Mitochondria

**Figure 4:**
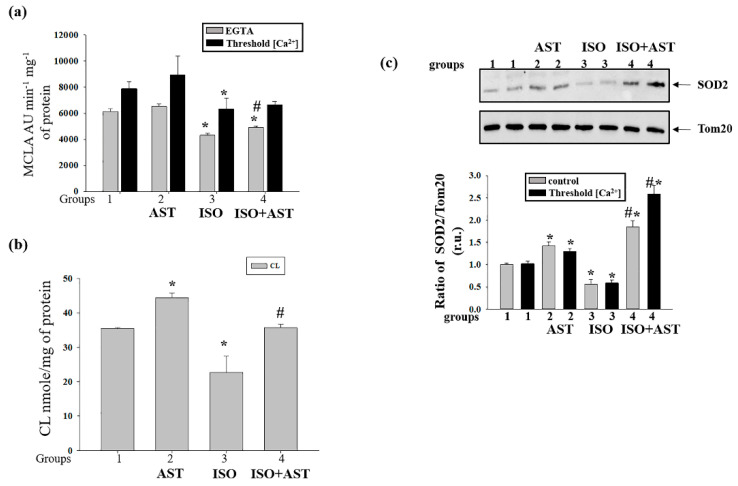
Effect of the administration of AST and injection of ISO on the concentrations of the superoxide anion, cardiolipin (CL) and Mn-SOD2 in rat heart mitochondria under mPTP opening. (**a**) Superoxide anion production in the presence of EGTA and threshold [Ca^2+^]; (**b**) alterations in the level of Mn-SOD2 under control and threshold [Ca^2+^] conditions; (**c**) alterations in the level of CL. * *p* < 0.05 indicates a significant difference in the protein level relative to the control (group 1). # *p* < 0.05 compared with RHM isolated from ISO-injected rats (group 3). The statistical significance of the differences between the pairs of the mean values was evaluated using an ANOVA type 2 (Student–Newman–Keuls) test.

Then, we assessed the production of ROS in RHM isolated from rats from each group (Figure 3 and Figure 4). In a medium supplemented with glutamate and malate, RHM from ISO-injected rats demonstrated significantly greater H_2_O_2_ production in both the absence (Figure 3a) and presence of Ca^2+^ (Figure 3b) compared with RHM from animals in the control group (RHM from group 3 vs. RHM from group 1). Contrary to our expectations, H_2_O_2_ production in RHM from AST-treated animals was highest among rats from all groups in the presence of both EGTA and Ca^2+^ (Figure 3a,b). Treatment with ISO strongly inhibited maximal H_2_O_2_ production by complex III (+ Antimycin A) by 40–50% (Figure 3c), whereas treatment with AST had no effect. In contrast, menadione stimulated H_2_O_2_ generation by initial segments of respiratory complexes I and II, and, presumably, mitochondrial diaphorase (+Menadione) was insignificantly affected by ISO treatment (Figure 3d). The consumption of AST did not cancel the effects of ISO on mitochondrial H_2_O_2_ production (Figure 3a–d). 

### 3.5. Effects of AST and ISO on Superoxide Anion Production, Expression of Mn-SOD2, and the Level of Cardiolipin in Rat Heart Mitochondria

The superoxide anion, in contrast to H_2_O_2_, penetrates poorly through the IMM. Therefore, SOD-sensitive MDCL in a mitochondrial suspension reflects either the generation of the superoxide anion in the outer Q-binding center of respiratory complex III or transient/permanent mPTP opening and the release of superoxide from the matrix (Figure 4a). The figure shows that treatment with AST slightly stimulated the release of superoxide from RHM in both the absence and presence of Ca^2+^ (insignificant increase). Treatment with ISO reduced the release of superoxide by ~30% and ~25% in the absence and presence of Ca^2+^, respectively. These changes could not be precluded by AST treatment. These data are in agreement with the suggestion that ISO inhibits ROS production by complex III. The fact that, in RHM from AST-consuming rats, only a minor increase in the release of the superoxide anion occurred while H_2_O_2_ production increased by 1.5–2 times may indicate a higher resistance of the mitochondria to mPTP opening.

It is well established that exposure of cells to ROS upregulates the production of the antioxidant defense enzymes [45]. In this study, we determined the content of Mn-SOD2 in RHM under experimental conditions (Figure 4b). Figure 4b (upper parts) shows a Western blot of SOD2 in RHM isolated from each group of rats. A quantitative analysis of the SOD2 level is shown in Figure 4b (lower parts). Protein bands were quantified after normalization with respect to Tom20. After treatment with AST, the SOD2 level increased by 43% and 27% in RHM when the mPTP was closed/open, respectively, compared with the control (RHM from group 2 vs. group 1). After injection of ISO, the content of SOD2 decreased by 45% and 42% when the mPTP was closed/open, respectively, in comparison with control RHM (RHM from group 3 vs. group 1). The combined actions of AST and ISO led to an increase in the SOD2 level of 85% relative to RHM from control rats when the mPTP was closed (RHM from group 4 vs. 1, grey columns) and by 2.5 times when the mPTP was open compared with RHM from rats injected with ISO (RHM from group 4 vs. group 1, black columns). It should be noted that the combination of AST and ISO increased the level of SOD2 by three times when the mPTP was closed (RHM from group 4 vs. 3, grey columns) and four times when the mPTP was open compared with the effect of ISO alone (RHM from group 4 vs. group 3, black columns). Therefore, the higher H_2_O_2_ production in RHM from AST-consuming rats may be, at least partially, due to a higher rate of conversion of the superoxide anion to H_2_O_2_ by SOD2. On the other hand, the fact that AST causes more intensive superoxide generation in the presence of a higher level of SOD2 suggests that AST stimulates superoxide generation in the mitochondria.

Cardiolipin, an ROS-sensitive phospholipid, is essential for the activity of respiratory chain complexes. We measured the level of CL in RHM isolated from rats of each group (Figure 4c). The CL level increased by 28% in RHM (45 vs. 35 nmol/mg protein) from rats to whom AST was administered relative to the control (RHM from group 2 vs. RHM from group 1). ISO injection decreased the content of CL in RHM by 30% to 23 nmol/mg protein compared with the control (RHM from group 3 vs. RHM from group 1). The administration of AST abolished the effect of ISO and preserved the CL level, which was 36 nmol/mg protein (RHM from group 4 vs. RHM from group 1). Hence, the exposure of rats to AST accelerates mitochondrial ROS production but, paradoxically, prevents the elimination of CL.

### 3.6. Effecst of AST and ISO on Calcium Retention Capacity and Membrane Potential in Rat Heart Mitochondria under mPTP Opening

**Figure 5:**
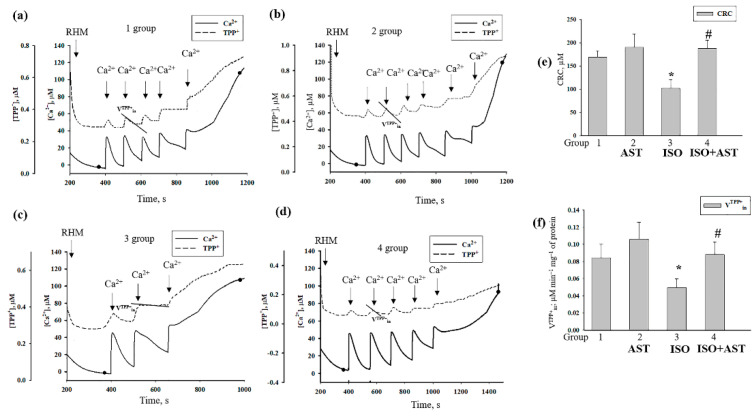
Effect of the administration of AST and injection of ISO on the Ca^2+^ retention capacity and membrane potential in rat heart mitochondria under mPTP opening.(**a**–**d**) Alterations in Ca^2+^ and TPP^+^ fluxes in rat heart mitochondria isolated from each group of rats; aliquots of mitochondrial suspension were taken at the points shown on curves a–d for further solubilization of Laemmli buffer and were subjected to SDS–PAGE and immunoblot analysis; (**e**) quantitative analysis of the Ca^2+^ capacity in rat heart mitochondria isolated from the rats of each group; (**f**) quantitative analysis of the rate of TPP^+^ influx in rat heart mitochondria isolated from the rats of each group. The data are presented as the means ± SDs of five independent experiments. * *p* < 0.05 indicates a significant difference in the protein level relative to the control (group 1). # *p* < 0.05 compared with RHM isolated from ISO-injected rats (group 3). The statistical significance of the differences between the pairs of mean values was evaluated using an ANOVA type 2 (Student–Newman–Keuls) test.

Because mitochondrial dysfunction induced by oxidative stress can affect the functional state in mitochondria [46], we examined the effects of mPTP opening induced in RHM by the administration of AST and injection of ISO on the membrane potential (Δψm), and the CRC in RHM isolated from each group of rats. Figure 5 a–d show the curves of changes in Ca^2+^ flow in RHM from the control group (group 1, Figure 5a), rats pretreated with AST (group 2, Figure 5b), rats injected with ISO (group 3, Figure 5c), and rats pretreated with AST and injected with ISO (group 4, Figure 5d). Ca^2+^ pulses (each 50 nmol per mg of protein) were added to mitochondria to reach the threshold Ca^2+^ concentration required for mPTP opening. In RHM from each group, the first addition of Ca^2+^ led to active accumulation of Ca^2+^ in the mitochondria. The release of accumulated Ca^2+^ (mPTP opening) occurred after the fifth Ca^2+^ pulse in RHM from group 1, the sixth Ca^2+^ pulse in RHM from group 2, the third Ca^2+^ pulse in RHM from group 3, and the fifth Ca^2+^ pulse in RHM from group 4. Figure 5e demonstrates the quantitative changes that occurred in the CRC in Ca^2+^-loaded RHM isolated from every experimental group of rats. We observed that AST administration increased the CRC by 15% in RHM compared with the control, while ISO injection decreased the CRC by 40%. The administration of AST in combination with ISO did not change the CRC in RHM from group 4 compared with RHM from group 1; however, under these conditions, the CRC increased by two times relative to RHM from ISO-injected rats (RHM from group 4 vs. group 3). A similar change was observed in the rate of TPP^+^ influx (Figure 5f). The rate of TPP^+^ influx decreased by two times in RHM from ISO-injected rats compared with the control group (RHM from group 3 vs. group 1). The administration of AST in combination with ISO increased the rate of TPP^+^ influx by 37.5% relative to this parameter in RHM from ISO-injected rats (RHM from group 4 vs. group 3) and did not change the rate of TPP^+^ influx compared to that in RHM from control rats (RHM from group 4 vs. group 1).

### 3.7. Effects of AST and ISO on Mitochondrial Swelling in Rat Heart Mitochondria 

**Figure 6:**
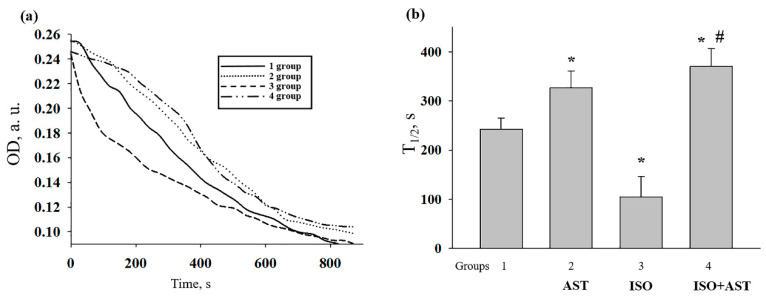
Effect of the administration of AST and injection of ISO on the swelling of rat heart mitochondria.(**a**) Curves of swelling of rat heart mitochondria isolated from rats of each group; (**b**) the half-maximum value of mitochondrial swelling (T_1/2_). The data are presented as the means ± SDs of five independent experiments. * *p* < 0.05 indicates a significant difference in the protein level relative to the control (group 1). # *p* < 0.05 compared with RHM isolated from ISO-injected rats (group 3). The statistical significance of the differences between the pairs of mean values was evaluated using an ANOVA type 2 (Student–Newman–Keuls) test.

Mitochondrial swelling is considered to be one of the characteristics of mPTP opening; therefore, we compared the swelling of RHM isolated from every group of rats (Figure 6). Figure 6a shows the representative curves of Ca^2+^-activated swelling of RHM isolated from each group of rats. Figure 6b demonstrates the average half-maximum (T_1/2_) value of mitochondrial Ca^2+^-activated swelling. The half-maximum value of the mitochondrial swelling of AST-treated rats increased by 30% (RHM from group 2 vs. group 1), whereas ISO decreased this parameter by 2.5 times compared with the control (RHM from group 3 vs. group 1). The administration of AST in combination with ISO increased the half-maximum value of mitochondrial swelling by 40% in comparison with the control (RHM from group 4 vs. group 1) and by 3.7 times relative to this parameter in RHM from ISO-injected rats (RHM from group 4 vs. group 3). These data support our suggestion that AST inhibits transient or sustainable mPTP opening despite the higher mitochondrial ROS production.

### 3.8. Effects of AST and ISO on the Concentration of mPTP Regulatory Proteins in Rat Heart Mitochondria 

**Figure 7:**
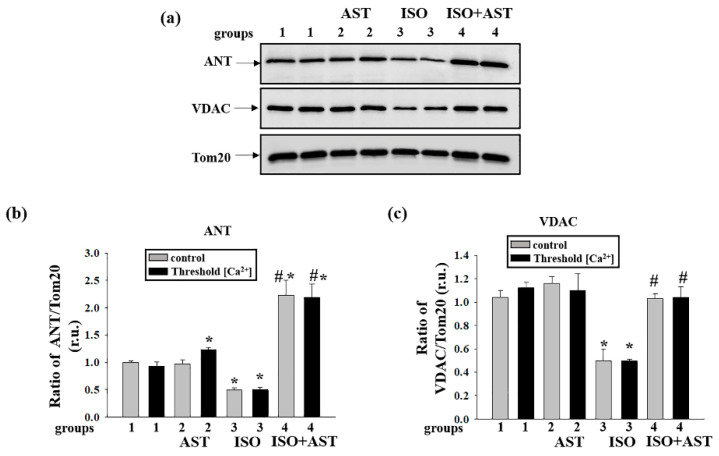
Effects of the administration of AST and injection of ISO on the content of the mitochondrial protein adenine nucleotide translocase (ANT) and the voltage-dependent anion channel (VDAC) in rat heart mitochondria isolated from rats from each group under mPTP opening. (**a**) Western blots stained with corresponding antibodies under control and threshold [Ca^2+^] conditions; (**b**) diagrams quantitatively reflecting changes in the ANT content in absolute units normalized to Tom20; (**c**) diagrams quantitatively reflecting changes in the level of VDAC in absolute units normalized to Tom20. The data are presented as the means ± SDs of three independent experiments. * *p* < 0.05 indicates a significant difference in the protein level relative to the control (group 1). # *p* < 0.05 compared to RHM isolated from ISO-injected rats (group 3). The statistical significance of the differences between the pairs of the mean values was evaluated using an ANOVA type 2 (Student–Newman–Keuls) test.

**Figure 8:**
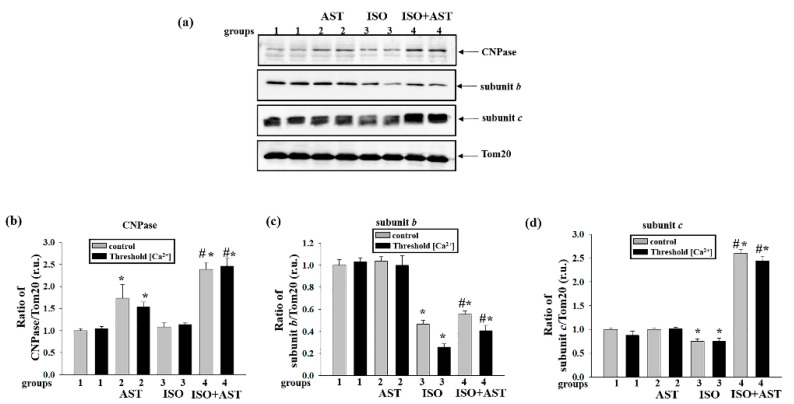
Effects of the administration of AST and injection of ISO on the content of the mitochondrial protein 2′, 3′-cyclic nucleotide 3′-phosphodiesterase (CNPase), subunit b, and subunit c in rat heart mitochondria isolated from rats of each group under mPTP opening. (**a**) Western blots stained with the corresponding antibodies under control and threshold [Ca^2+^] conditions; (**b**) diagrams quantitatively reflecting changes in the CNPase content in absolute units normalized to Tom20; (**c**) diagrams quantitatively reflecting changes in the level of subunit b in absolute units normalized to Tom20; (**d**) diagrams quantitatively reflecting changes in the level of subunit c in absolute units normalized to Tom20. The data are presented as the means ± SDs of three independent experiments. * *p* < 0.05 indicates a significant difference in the protein level relative to the control (group 1). # *p* < 0.05 compared with RHM isolated from ISO-injected rats (group 3). The statistical significance of the differences between the pairs of mean values was evaluated using an ANOVA type 2 (Student–Newman–Keuls) test.

It is known that regulatory proteins play an important role in the mPTP; therefore, we examined how the administration of AST and ISO influences the concentrations of proteins such as ANT, VDAC, subunit c, CNPase, and subunit b of ATP synthase in RHM isolated from each group of rats under conditions of mPTP opening (Figure 7 and Figure 8). Figure 7a shows Western blots of ANT and VDAC in RHM isolated from each group of rats. A quantitative analysis of protein levels is shown in Figure 7b,c. Protein bands were quantified after normalization with respect to Tom20. As seen from Figure 7b, the level of ANT decreased by 50% and 45% when the mPTP was closed and open, respectively, in RHM isolated from the ISO-injected rats compared with the control group of rats (RHM from group 3 vs. group 1). It is interesting to note that the administration of AST increased the level of ANT by two times when the mPTP was closed or open relative to the control (RHM from group 4 vs. group 1) and four times compared to the actions of ISO alone (RHM from group 4 vs. group 3) when the mPTP was closed or open. The content of VDAC diminished in RHM from ISO-injected rats when the mPTP was closed and open by 50% and 60%, respectively, relative to the control (RHM from group 3 vs. group 1). The combined effect of AST and ISO increased the concentration of VDAC by two times when the mPTP was closed or open compared with RHM from ISO-injected rats (RHM from group 4 vs. group 3). AST abolished the effect induced by ISO and increased the concentrations of ANT and VDAC in RHM.

At the next step, we examined the influences of AST and ISO on the concentrations of CNPase, subunit b, and subunit c in RHM isolated from each group of rats (Figure 8). Figure 8a shows Western blots of CNPase, subunit b, and subunit c in RHM. A quantitative analysis of protein levels is shown in Figure 8b–d.

Figure 8b shows that AST upregulated the CNPase concentration when the mPTP was closed or open by 70% and 50%, respectively, compared with the control (RHM from group 2 vs. group 1). It should be noted that AST in combination with ISO increased the level of CNPase when the mPTP was closed or open by two times compared with the control (RHM from group 4 vs. group 1) and by two times relative to the effect of ISO alone (RHM from group 4 vs. group 3).

Figure 8c shows that when the mPTP was closed and open, the concentration of subunit b decreased in RHM isolated from ISO-injected rats by 50% and 75%, respectively, compared with the control (RHM from group 3 vs. group 1). AST in combination with ISO decreased the concentration of subunit b when the mPTP was closed or open by 45% and 60% compared with the control (RHM from group 4 vs. group 1) and increased it by 20% and 40% relative to the effect of ISO alone (RHM from group 4 vs. group 3).

Similar changes were observed in the concentration of subunit c (Figure 8d). ISO injection diminished the concentration of subunit c, irrespective of whether the mPTP was closed or open, by 25% and 14%, respectively (RHM from group 3 vs. group 1). Under these conditions (mPTP closed/open), AST increased the concentration of subunit *c* by more than 2.5 times in RHM after ISO injection relative to the control (RHM from group 4 vs. group 1) and by more than three times compared with the effect of ISO alone (RHM from group 4 vs. group 3).

## 4. Discussion

Mitochondria play an important role in the normal functioning of the heart as well as in the pathogenesis and development of various heart diseases. Mitochondrial dysfunction resulting from heart damage is crucial for the development of these disorders [21]. It is important for normal cell functioning that the structural and functional integrity of mitochondria are supported, since mitochondria play leading roles in energy metabolism, maintaining the redox state of cells, and regulating apoptosis. Since mitochondria are the main source of ROS, their dysfunction causes oxidative stress, which can lead to cell death [2]. Therefore, it is important to find means and approaches to preventing the development of heart disorders.

AST is naturally produced by some marine bacteria, algae, and plants. Many red-colored marine animals (e.g., salmons, shrimps) and a few bird species (e.g., flamingo) receive AST orally through the food chain [47]. As a potent antioxidant, AST has been used as a common antioxidant agent in research and as a dietary supplement intended for human, animal, and aquaculture consumption. AST has been found to have numerous health benefits in humans including improvement in the condition of patients with cardiovascular failure and enhancement of the immune response [48].

In the present work, we carried out a histological analysis of tissue sections of the left ventricle of the heart to reveal the lesions caused by the injection of ISO. The analysis showed that AST can significantly reduce both degeneration and edema of heart muscle fibers and the degree of fibrous myocardial damage after ISO injection. The data obtained using digital bioimaging of transmural histograms of LV tissue from animals of all groups suggested that a significant decrease in the degree of fibrosis of subendocardial heart lesions had occurred, demonstrating the protective effect of AST.

Earlier, we showed that the pretreatment of rats with AST improves the functional state of the mitochondria by increasing the RCI and activity of respiratory chain complexes and ATP synthase in RHM injured by ISO [33]. Here, we used the same concentration of ISO and found that ISO injection decreased the RCI in RHM, whereas AST abolished the effect of ISO and increased the RCI in RHM.

It should be stressed that the protective effect of AST in ISO-treated rats, presumably, may not be ascribed exclusively to its antioxidant properties. Indeed, AST itself stimulated hydrogen peroxide and superoxide anion production and increased the concentration of oxidative-stress-responsive SOD2 (see Figure 4 and Figure 5). A similar effect of AST was shown for cancer [49]. Partial prevention of elimination of the components of the damaged respiratory chain complexes in ISO-injected rats (Figure 2) may be related to the stimulation of mitochondrial biogenesis by AST [50].

At the same time, AST, being a polar carotenoid, is known to preserve the structure of membranes and prevent their disorganization and the penetration of oxygen into the lipid bilayer [27]. Together with the high antioxidant activity of AST, this can explain the increase in the CL content following elevated ROS production (Figure 3 and Figure 4). In addition, the partial inhibition of the oxygen consumption rate in state 3 in RHM from AST-consuming rats (see Table 1) can be explained either by inhibition of the rate of oxygen penetration through the compact lipid membrane or by a “proton trap” by CL whose steady-state concentration increased following the application of AST [51].

The phospholipid cardiolipin is located in the IMM [15]. The oxidation of CL results in dysfunction of the mitochondrial ETC and is believed to promote the release of pro-apoptotic proteins, including cytochrome *c* [52,53], from the intermembrane space and, therefore, the formation of the mPTP in mitochondria [54]. The respiratory chain is composed of five complexes, which are responsible for electron transport to molecular oxygen. Electron transport is coupled with proton export across the IMM, generating the membrane potential, which acts as an energy source for the synthesis of ATP by ATP synthase. CL plays a role in the development of structural integrity and enzymatic activity in these complexes. There are specific binding sites for CL in Complex I [55], Complex III [56], Complex IV [57], and Complex II [58]. We suggest that a decrease in the CL level (Figure 4) in RHM from ISO-injected rats can diminish the concentrations of basic subunits from the ETC complexes (Figure 2) and, hence, disturb the functional state of the mitochondria (Table 1). Since CL is involved in the opening of the mPTP, the CRC of RHM from ISO-injected rats decreased (Figure 5), which accelerated the opening of the mPTP and increased the rate of mitochondrial swelling (Figure 6). AST improved the functional state of RHM from ISO-injected rats, while the level of CL increased, which led to increases in the RCI and the Ca^2+^ capacity and decelerated mitochondrial swelling. Antioxidants provide a protective mechanism that destroys harmful ROS and inhibits lipid peroxidation. Protective enzymes, such as catalase, glutathione peroxidase, and superoxide dismutase, scavenge free radicals, help to restore the antioxidant defense system, and inhibit ROS production [59]. Here, the level of Mn-SOD2 was diminished by ISO injection in RHM, whereas pretreatment with AST increased the content of the enzyme in RHM isolated after ISO injection.

ANT is a protein of the IMM that is involved in the exchange of extramitochondrial ADP and intramitochondrial ATP, is coupled to oxidative phosphorylation, and affects the mitochondrial membrane [60]. Furthermore, ANT plays a leading role in cell survival because it regulates mPTP opening, thereby affecting mitochondrial integrity and cell death [11]. We revealed that ISO caused a decrease in the ANT level, which was accompanied by acceleration of mPTP opening and mitochondrial swelling, whereas AST inhibited this effect, increasing the content of ANT and the Ca^2+^ capacity and decelerating mitochondrial swelling. Another regulator of the mPTP is VDAC [7]. Being localized in the OMM, it regulates its permeabilization [61]. Despite the fact that VDAC is not a structural component of the pore, it is involved in its regulation [62]. The decreased level of VDAC in RHM from ISO-injected rats may be a reason for changes in the sensitivity to Ca^2+^ upon activation of the mPTP and the acceleration of mitochondrial swelling. AST increased the content of VDAC in RHM from ISO-injected rats and the Ca^2+^ capacity and inhibited the swelling of RHM.

Earlier we identified 2′,3′-cyclic nucleotide-3′-phosphodiestarase (CNPase) in rat brain mitochondria [13]. This myelin protein has been found in non-myelin tissues; it is localized in the OMM and mitoplasts [14]. One function of the protein is its participation in the regulation of mPTP [13]. In addition, CNPase is co-localized with ANT and VDAC [14,63]. In the present study, we found that the level of CNPase diminished in RHM isolated from ISO-injected rats, whereas the application of AST in combination with ISO increased the content of CNPase when the mPTP was closed/open. A similar effect was observed with both VDAC and ANT.

Subunit *c*, a mitochondrial *N*,*N*–dicyclohexylcarbodiimide (DCCD)-binding proteolipid, and subunit *b* are the subunits of the F_o_ sector of ATP synthase [64,65]. Earlier, we established that subunit *c* of ATP synthase is a structural and/or regulatory component of the mPTP complex whose activity might be modulated by calcium-dependent phosphorylation [12]. Moreover, Neginskaya and coauthors showed that subunit *c* of ATP synthase plays a critical role in the formation of the native calcium-induced mPTP channel [66]. The levels of subunit *b* (Figure 8) and the alpha subunit (ATP5A in Figure 2) of sector F_1_ of ATP synthase decreased in RHM isolated from ISO-injected rats, whereas AST increased the levels of these subunits and abolished the effect of ISO. It is known that ATP synthase catalyzes the final coupling step of oxidative phosphorylation to supply energy in the form of ATP. Alterations at this stage can strongly influence mitochondrial respiration and, hence, heart functioning. It is well documented that cardiac contractility depends on mitochondria and that a reduction in the content of myocardial ATP is a distinguishing sign of heart failure [65]. In the present work, ISO injection induced decreases in the levels of subunits *c* and *b* as well as the basic subunits of the respiratory chain complexes and in the RCI of RHM, while AST abolished the inductive effect of ISO and raised both the concentrations of the subunits and the RCI value in RHM.

## 5. Conclusions

In summary, AST reduced both the degeneration and edema of heart muscle fibers as well as the degree of fibrous myocardial damage after ISO injection. ISO severely decreased the respiratory capacity of RHM, while pretreatment with AST restored both respiration and mitochondrial coupling. ISO caused a strong decline in the concentrations of subunits of all respiratory chain complexes and ATP synthase. Though pretreatment with AST alone had no effect on the concentrations of the subunits of respiratory complexes, it reduced the ISO-dependent decline in their level by two to three times. AST administration accelerated ROS production; however, it prevented the elimination of CL, which plays an important role in the regulation of membrane integrity and the activity of respiratory chain complexes. The pretreatment of rats with AST inhibited mPTP opening, despite the high production of ROS in mitochondria. Presumably, this inhibition occurred due to the fact that AST increased the level of mPTP regulatory proteins when the mPTP was closed or open in RHM isolated from rats injected with ISO. All of the aforesaid results suggest that AST exhibits protective properties in mitochondria and can be used to prevent the development of cardiovascular diseases.

## Figures and Tables

**Table 1:** Effect of AST administration and ISO injection on respiratory activity in RHM isolated from rats from each group. Respiratory activity rates in states 2–4 were defined as the number of ng-O atoms consumed by the mitochondria per minute per mg of protein. The respiratory control index (RCI) was calculated as the ratio of V_st.3_ to V_st.4_. * *p* < 0.05 indicates a significant difference in the protein level relative to the control (group 1). ^#^ *p* < 0.05 compared with rat heart mitochondria (RHM) isolated from ISO-injected rats (group 3). The statistical significance of the differences between the pairs of mean values was evaluated using an ANOVA type 2 (Student–Newman–Keuls) test.

	V_St.2_	V_St.__3_	V_St.4_	RCI
RHM group 1	7.55 ± 0.32	40.91 ± 345	7.39 ± 0.57	5.21 ± 0.52
RHM group 2	8.48 ± 0.94	40.10 ± 5.08	7.32 ± 1.49	5.60 ± 0.73
RHM group 3	4.82 ± 0.82 *	17.26 ± 1.10 *	6.76 ± 0.52	2.52 ± 0.73 *
RHM group 4	6.74 ± 0.64	41.27 ± 3.09 ^#^	8.67 ± 0.91 ^#^	4.8 ± 0.88 ^#^

## Supplementary Materials

The following are available online at https://www.mdpi.com/2227-9059/8/10/437/s1, Figure S1: The statistical significance of the differences between the pairs of mean values was evaluated using the Student-Newman-Keul test (Figure 1, Figure 2, Figure 3, Figure 4, Figure 5, Figure 6, Figure 7 and Figure 8).
