# Supplementary material for: Isoproterenol-Induced Permeability Transition Pore-Related Dysfunction of Heart Mitochondria Is Attenuated by Astaxanthin
Source: Biomedicines. 2020 Oct 20;8(10):437. doi: 10.3390/biomedicines8100437 (PMC7589423; doi:10.3390/biomedicines8100437)
Supplement: Supplementary file 1 [file biomedicines-08-00437-s001.pdf]

To Fig 1.

Histology

	group 1	group 2	group 3	group 4
Mean 1	**-2.3625**	**-0.7098**	**30.3102**	**11.5002**
Mean 2	**-0.7715**	**-3.7087**	**23.664**	**8.401**
Mean 3	**1.8492**	**-3.4398**	**28.1929**	**9.4729**
Mean 4	**0.9683**	**-4.181**	**17.0851**	**8.5077**
Mean 5	**0.3163**	**-1.2238**	**32.0199**	**18.8683**

One Way Analysis of Variance All Pairwise Multiple Comparison Procedures (Student-Newman-Keuls Method) :

Comparison	Diff of Means	P<0.050
Group 2 vs. Group 1	2.653	No
Group 3 vs. Group 1	26.254	Yes
Group 4 vs. Group 1	11.35	Yes
Group 4 vs. Group 3	14.904	Yes

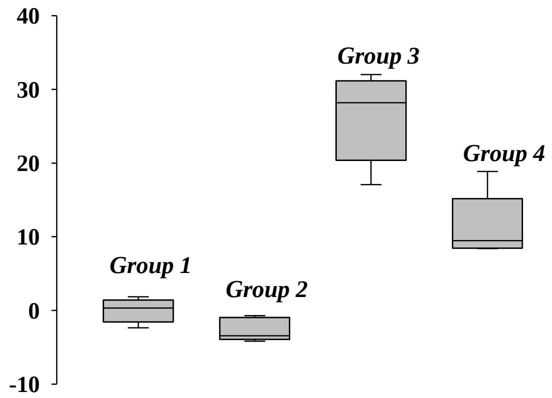

To Fig 2.

CV **b**

	Group 1 (w/o adds)		Group 2 (AST)		Group 3 (ISO)		Group 4 (ISO+AST)	
	Control Col 1	$+Ca^{2+}$ Col 2	Control Col 3	$+Ca^{2+}$ Col 4	Control Col 5	$+Ca^{2+}$ Col 6	Control Col 7	$+Ca^{2+}$ Col 8
Mean 1	0.9445	0.8957	0.9662	0.7402	0.4346	0.454	0.8182	0.8822
Mean 2	0.9653	0.9741	1.015	0.7833	0.4498	0.3593	0.7779	0.9534
Mean 3	1.0897	0.8979	1.084	0.9576	0.5369	0.492	0.8303	0.9817

CIII **c**

	Group 1 (w/o adds)		Group 2 (AST)		Group 3 (ISO)		Group 4 (ISO+AST)	
	Control Col 1	$+Ca^{2+}$ Col 2	Control Col 3	$+Ca^{2+}$ Col 4	Control Col 5	$+Ca^{2+}$ Col 6	Control Col 7	$+Ca^{2+}$ Col 8
Mean 1	1.0162	0.9729	0.8654	0.8373	0.4418	0.4702	0.8438	1.0474
Mean 2	0.9822	0.9617	0.8656	1.0032	0.3551	0.3408	0.8487	0.9309
Mean 3	1.0017	1.0917	0.9699	1.0917	0.3931	0.4618	0.9307	1.0997

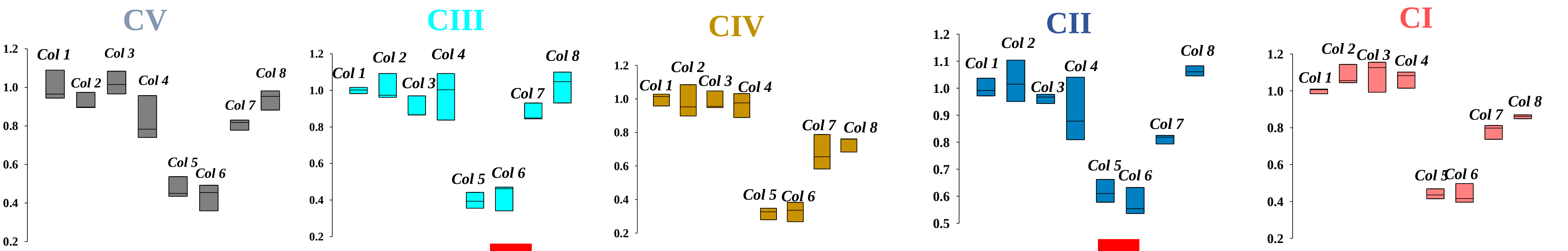

CIV **d**

	Group 1 (w/o adds)		Group 2 (AST)		Group 3 (ISO)		Group 4 (ISO+AST)	
	Control Col 1	$+Ca^{2+}$ Col 2	Control Col 3	$+Ca^{2+}$ Col 4	Control Col 5	$+Ca^{2+}$ Col 6	Control Col 7	$+Ca^{2+}$ Col 8
Mean 1	1.0283	1.0846	1.0473	1.0315	0.3268	0.3833	0.655	0.7602
Mean 2	0.9578	0.8982	0.9551	0.889	0.2793	0.2675	0.7878	0.7615
Mean 3	1.014	0.9523	0.9478	0.9759	0.3484	0.3363	0.5822	0.683

CII **e**

	Group 1 (w/o adds)		Group 2 (AST)		Group 3 (ISO)		Group 4 (ISO+AST)	
	Control Col 1	$+Ca^{2+}$ Col 2	Control Col 3	$+Ca^{2+}$ Col 4	Control Col 5	$+Ca^{2+}$ Col 6	Control Col 7	$+Ca^{2+}$ Col 8
Mean 1	1.0368	0.9506	0.9771	1.0409	0.6102	0.5537	0.8189	1.0455
Mean 2	0.9715	1.0149	0.9432	0.809	0.5778	0.5362	0.8254	1.0827
Mean 3	0.9917	1.1039	0.9671	0.8781	0.6623	0.6323	0.7933	1.0603

f CI

	Group 1 (w/o adds)		Group 2 (AST)		Group 3 (ISO)		Group 4 (ISO+AST)	
	Control Col 1	$+Ca^{2+}$ Col 2	Control Col 3	$+Ca^{2+}$ Col 4	Control Col 5	$+Ca^{2+}$ Col 6	Control Col 7	$+Ca^{2+}$ Col 8
Mean 1	1.0064	1.0552	0.9925	1.0145	0.4353	0.497	0.7978	0.8491
Mean 2	1.0097	1.1435	1.1261	1.0826	0.4145	0.4157	0.7374	0.8616
Mean 3	0.9838	1.0445	1.1554	1.1019	0.4686	0.3958	0.8122	0.8698

To Fig 2.

One Way Analysis of Variance All Pairwise Multiple Comparison Procedures (Student-Newman-Keuls Method) :

b CV

Comparison	Diff of Means	P<0.050
Col 3 vs. Col 1	0.0219	No
Col 5 vs. Col 1	0.526	Yes
Col 7 vs. Col 1	0.191	Yes
Col 7 vs. Col 5	0.335	Yes
Col 4 vs. Col 2	0.0956	No
Col 6 vs. Col 2	0.487	Yes
Col 8 vs. Col 2	0.0165	No
Col 8 vs. Col 6	0.504	Yes

c CIII

Comparison	Diff of Means	P<0.050
Col 3 vs. Col 1	0.0997	No
Col 5 vs. Col 1	0.603	Yes
Col 7 vs. Col 1	0.126	Yes
Col 7 vs. Col 5	0.478	Yes
Col 4 vs. Col 2	0.0314	No
Col 6 vs. Col 2	0.585	Yes
Col 8 vs. Col 2	0.0172	No
Col 8 vs. Col 6	0.602	Yes

d CIV

Comparison	Diff of Means	P<0.050
Col 3 vs. Col 1	0.0167	No
Col 5 vs. Col 1	0.682	Yes
Col 7 vs. Col 1	0.325	Yes
Col 7 vs. Col 5	0.357	Yes
Col 4 vs. Col 2	0.0129	No
Col 6 vs. Col 2	0.649	Yes
Col 8 vs. Col 2	0.243	Yes
Col 8 vs. Col 6	0.406	Yes

e CII

Comparison	Diff of Means	P<0.050
Col 3 vs. Col 1	0.0375	No
Col 5 vs. Col 1	0.383	Yes
Col 7 vs. Col 1	0.187	Yes
Col 7 vs. Col 5	0.196	Yes
Col 4 vs. Col 2	0.114	No
Col 6 vs. Col 2	0.449	Yes
Col 8 vs. Col 2	0.0397	No
Col 8 vs. Col 6	0.243	Yes

f CI

Comparison	Diff of Means	P<0.050
Col 3 vs. Col 1	0.0914	No
Col 5 vs. Col 1	0.56	Yes
Col 7 vs. Col 1	0.218	Yes
Col 7 vs. Col 5	0.343	Yes
Col 4 vs. Col 2	0.0148	No
Col 6 vs. Col 2	0.645	Yes
Col 8 vs. Col 2	0.221	Yes
Col 8 vs. Col 6	0.424	Yes

To Fig 3.

a EGTA

b Ca^{2+}

c Antimycin A

Menadion

EGTA:

	group 1	group 2	group 3	group 4
Mean 1	153.254	155.076	154.2425	153.985
Mean 2	211.32	247.8493	201.1665	247.021
Mean 3	168.2341	180.2	175.591	187.24
Mean 4	178.2	222.3907	156.8632	200.2

Ca^{2+}:

	group 1	group 2	group 3	group 4
Mean 1	221.1721	468.3049	349.2489	362.6013
Mean 2	218.4544	348.2448	303.0058	272.8328
Mean 3	219.2125	421.1324	311.5495	300.8622
Mean 4	215.022	397.0241	345.52	335.2542

Antimycin A:

	group 1	group 2	group 3	group 4
Mean 1	3065.839	2675.2	1257.54	1738.745
Mean 2	3034.017	2233.789	1998.255	1283.949
Mean 3	2287.21	2721.132	1311.515	1750.334
Mean 4	2157.54	2297.024	1045.52	1274.35

Menadion:

	group 1	group 2	group 3	group 4
Mean 1	3065.839	2675.2	1257.54	1738.745
Mean 2	3034.017	2233.789	1998.255	1283.949
Mean 3	2287.21	2721.132	1311.515	1750.334
Mean 4	2157.54	2297.024	1045.52	1274.35

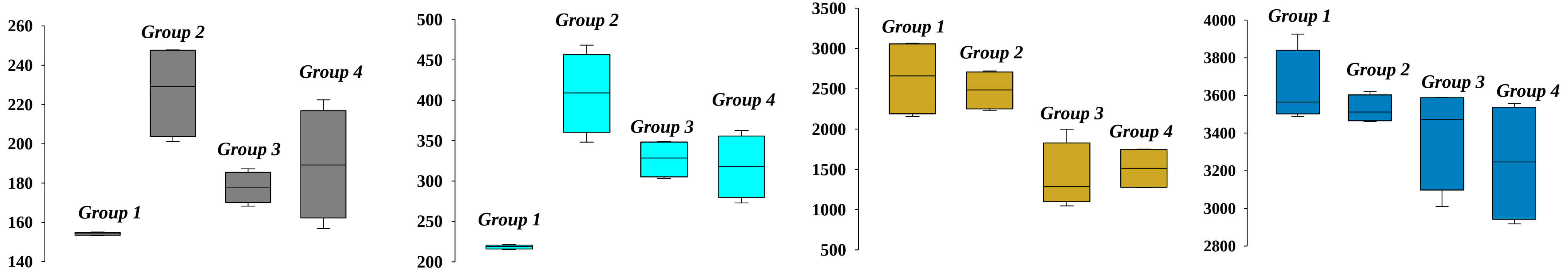

EGTA

Comparison	Diff of Means	P<0.050
Group 2 vs. Group 1	72.7	Yes
Group 3 vs. Group 1	23.677	Yes
Group 4 vs. Group 1	35.274	No
Group 4 vs. Group 3	11.597	No

Ca^{2+}

Comparison	Diff of Means	P<0.050
Group 2 vs. Group 1	190.211	Yes
Group 3 vs. Group 1	108.866	Yes
Group 4 vs. Group 1	99.422	No
Group 4 vs. Group 3	9.443	No

Antimycin A

Comparison	Diff of Means	P<0.050
Group 2 vs. Group 1	154.365	No
Group 3 vs. Group 1	1232.944	Yes
Group 4 vs. Group 1	1124.307	Yes
Group 4 vs. Group 3	108.637	No

Menadion

Comparison	Diff of Means	P<0.050
Group 2 vs. Group 1	-	No
Group 3 vs. Group 1	-	No
Group 4 vs. Group 1	-	No
Group 4 vs. Group 3	-	No

To Fig 4.

	Group 1 (w/o adds)		Group 2 (AST)		Group 3 (ISO)		Group 4 (ISO+AST)	
	EGTA Col 1	$+Ca^{2+}$ Col 2	EGTA Col 3	$+Ca^{2+}$ Col 4	EGTA Col 5	$+Ca^{2+}$ Col 6	EGTA Col 7	$+Ca^{2+}$ Col 8
Mean 1	5967.334	6669.512	4054.999	5091.304	8475.932	7761.909	7527.677	6831.394
Mean 2	6296.909	6381.048	4306.862	4947.647	7500.807	10961.18	5521.749	6484.883
Mean 3	6425.54	6785.24	4287.014	4789.665	7147.54	10012.36	6011.64	6875.65
Mean 4	5987.35	6375.001	4585.015	4778.644	8201.225	7541.35	6247.465	6587.001

	group 1	group 2	group 3	group 4
Mean 1	35.2	45.45	30.5	35.71
Mean 2	35.7	42.6	22.7	37.7
Mean 3	34.9	46	19	34.8
Mean 4	35.9	43.9	18.9	34.9

c

SOD2

	Group 1 (w/o adds)		Group 2 (AST)		Group 3 (ISO)		Group 4 (ISO+AST)	
	Control Col 1	$+Ca^{2+}$ Col 2	Control Col 3	$+Ca^{2+}$ Col 4	Control Col 5	$+Ca^{2+}$ Col 6	Control Col 7	$+Ca^{2+}$ Col 8
Mean 1	1.0395	0.9847	1.3316	1.3107	0.5844	0.6426	1.9294	2.6305
Mean 2	0.9677	1.0954	1.4384	1.3609	0.6614	0.6148	1.9369	2.3655
Mean 3	0.993	0.9849	1.505	1.2295	0.4472	0.5108	1.6923	2.7463

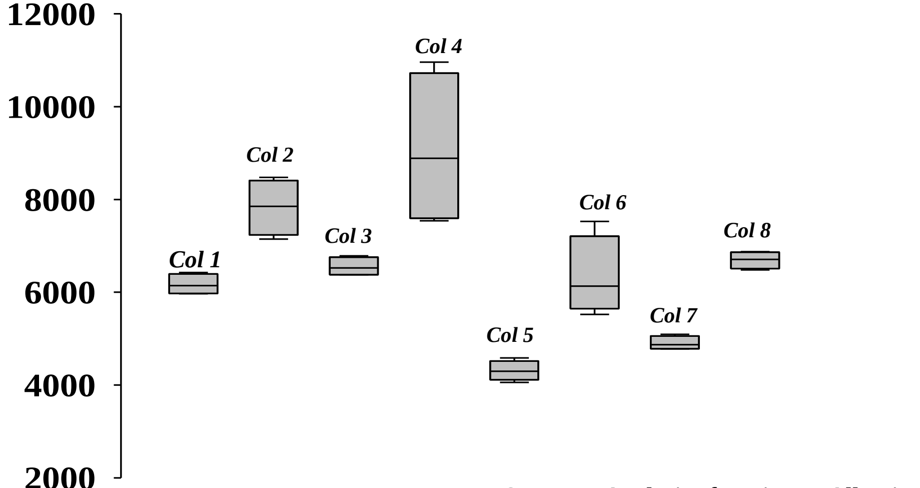

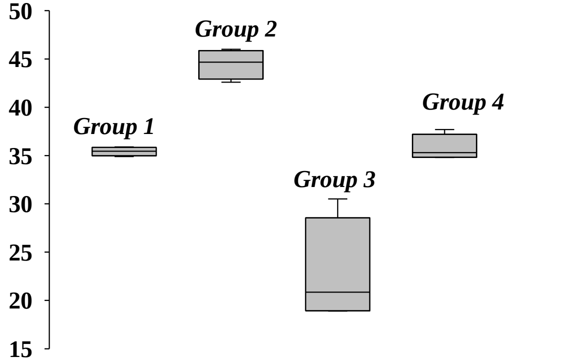

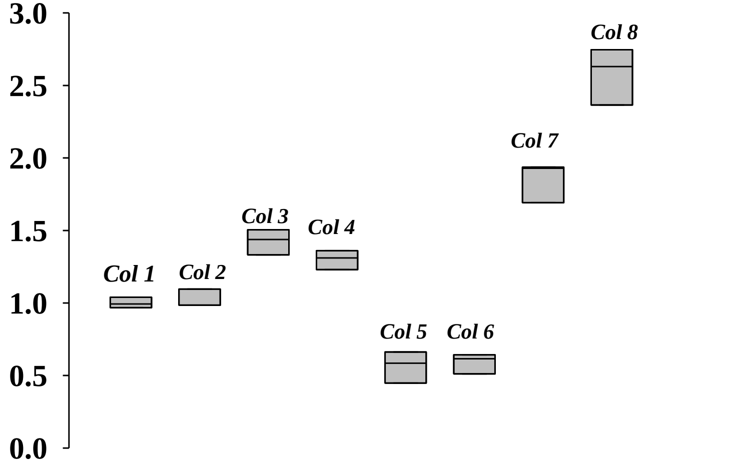

One Way Analysis of Variance All Pairwise Multiple Comparison Procedures (Student-Newman-Keuls Method) :

Comparison	Diff of Means	P<0.050
Col 3 vs. Col 1	383.417	No
Col 5 vs. Col 1	1860.811	Yes
Col 7 vs. Col 1	1267.468	Yes
Col 7 vs. Col 5	593.343	Yes
Col 4 vs. Col 2	1237.824	No
Col 6 vs. Col 2	1504.243	Yes
Col 8 vs. Col 2	1136.644	Yes
Col 8 vs. Col 6	367.599	No

Comparison	Diff of Means	P<0.050
Group 2 vs. Group 1	9.063	Yes
Group 3 vs. Group 1	12.650	Yes
Group 4 vs. Group 1	0.353	No
Group 4 vs. Group 3	13.003	Yes

Comparison	Diff of Means	P<0.050
Col 3 vs. Col 1	0.425	Yes
Col 5 vs. Col 1	0.436	Yes
Col 7 vs. Col 1	0.853	Yes
Col 7 vs. Col 5	1.289	Yes
Col 4 vs. Col 2	0.279	Yes
Col 6 vs. Col 2	0.432	Yes
Col 8 vs. Col 2	1.559	Yes
Col 8 vs. Col 6	1.991	Yes

To Fig 5.

e

CRC

	group 1	group 2	group 3	group 4
Mean 1	150	215	50	200
Mean 2	185	150	150	170
Mean 3	160	220	80	170
Mean 4	170	190	130	190
Mean 5	180	180	100	210

f

V^{TPP+}

	group 1	group 2	group 3	group 4
Mean 1	0.07	0.15	0.04	0.09
Mean 2	0.07	0.11	0.03	0.11
Mean 3	0.11	0.09	0.05	0.07
Mean 4	0.09	0.08	0.07	0.08
Mean 5	0.08	0.1	0.058	0.09

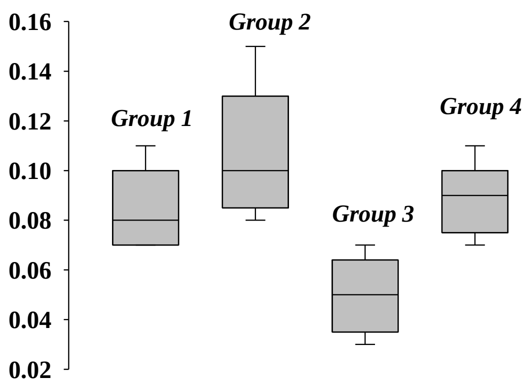

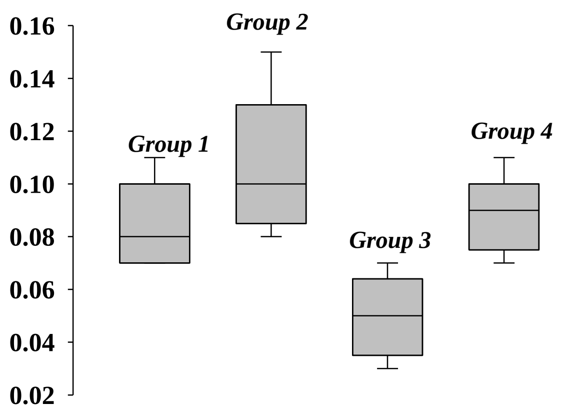

One Way Analysis of Variance All Pairwise Multiple Comparison Procedures (Student-Newman-Keuls Method) :

Comparison	Diff of Means	P<0.050
Group 2 vs. Group 1	22	No
Group 3 vs. Group 1	67	Yes
Group 4 vs. Group 1	19	No
Group 4 vs. Group 3	67	Yes

Comparison	Diff of Means	P<0.050
Group 2 vs. Group 1	0.022	No
Group 3 vs. Group 1	0.0344	Yes
Group 4 vs. Group 1	0.004	No
Group 4 vs. Group 3	0.0384	Yes

To Fig 6.

Swelling $T_{1/2}$

	group 1	group 2	group 3	group 4
Mean 1	207	310	61	326
Mean 2	254	335	65	393
Mean 3	262	365	124	411
Mean 4	246	282	106	355
Mean 5	262	305	102	336

One Way Analysis of Variance All Pairwise Multiple Comparison Procedures (Student-Newman-Keuls Method) :

Comparison	Diff of Means	P<0.050
Group 2 vs. Group 1	6.072	Yes
Group 3 vs. Group 1	137.833	Yes
Group 4 vs. Group 1	127.5	Yes
Group 4 vs. Group 3	265.333	Yes

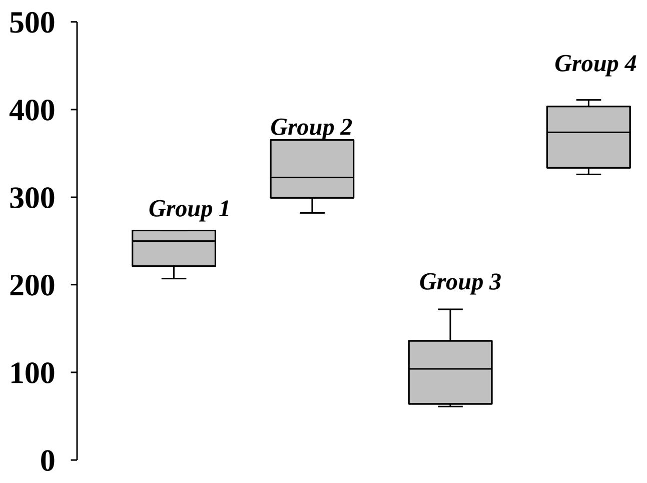

To Fig 7.

b **ANT** c **VDAC**

	ANT Group 1 (w/o adds)		ANT Group 2 (AST)		ANT Group 3 (ISO)		ANT Group 4 (ISO+AST)		VDAC Group 1 (w/o adds)		VDAC Group 2 (AST)		VDAC Group 3 (ISO)		VDAC Group 4 (ISO+AST)	
	Control Col 1	$+Ca^{2+}$ Col 2	Control Col 3	$+Ca^{2+}$ Col 4	Control Col 5	$+Ca^{2+}$ Col 6	Control Col 7	$+Ca^{2+}$ Col 8	Control Col 1	$+Ca^{2+}$ Col 2	Control Col 3	$+Ca^{2+}$ Col 4	Control Col 5	$+Ca^{2+}$ Col 6	Control Col 7	$+Ca^{2+}$ Col 8
Mean 1	1.0039	0.8421	0.9488	1.276	0.4563	0.4475	1.9193	1.9456	1.0236	1.0875	1.1129	0.9503	0.3838	0.4844	1.0717	1.12
Mean 2	1.0197	0.9655	1.0534	1.2078	0.5333	0.5344	2.4225	2.4364	1.1042	1.1775	1.2265	1.2327	0.5788	0.4956	0.986	1.0601
Mean 3	0.9764	0.9739	0.8974	1.2017	0.4752	0.5162	2.3444	2.1884	0.992	1.1004	1.1448	1.1183	0.5232	0.5134	1.0339	0.9432

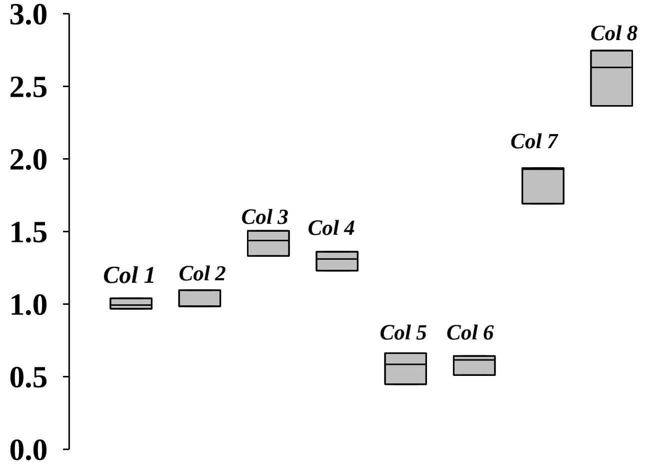

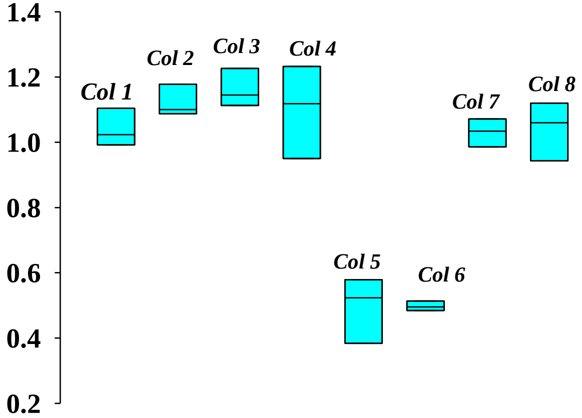

One Way Analysis of Variance All Pairwise Multiple Comparison Procedures (Student-Newman-Keuls Method) :

ANT

Comparison	Diff of Means	P<0.050
Col 3 vs. Col 1	0.0335	No
Col 5 vs. Col 1	0.512	Yes
Col 7 vs. Col 1	1.229	Yes
Col 7 vs. Col 5	1.74	Yes
Col 4 vs. Col 2	0.301	Yes
Col 6 vs. Col 2	0.428	Yes
Col 8 vs. Col 2	1.263	Yes
Col 8 vs. Col 6	1.691	Yes

VDAC

Comparison	Diff of Means	P<0.050
Col 3 vs. Col 1	0.121	No
Col 5 vs. Col 1	0.545	Yes
Col 7 vs. Col 1	0.00938	No
Col 7 vs. Col 5	0.535	Yes
Col 4 vs. Col 2	0.0214	No
Col 6 vs. Col 2	0.624	Yes
Col 8 vs. Col 2	0.0807	No
Col 8 vs. Col 6	0.543	Yes

To Fig 8.

b **CNPase**

	Group 1 (w/o adds)		Group 2 (AST)		Group 3 (ISO)		Group 4 (ISO+AST)	
	Control Col 1	$+Ca^{2+}$ Col 2	Control Col 3	$+Ca^{2+}$ Col 4	Control Col 5	$+Ca^{2+}$ Col 6	Control Col 7	$+Ca^{2+}$ Col 8
Mean 1	1.0441	1.0915	1.5853	1.491	1.003	1.0985	2.5589	2.5631
Mean 2	0.9637	1.0139	2.0862	1.6664	1.1881	1.1296	2.3572	2.5647
Mean 3	0.9923	1.0344	1.5254	1.4636	1.0609	1.1807	2.2458	2.2465

c **Sub b**

	Group 1 (w/o adds)		Group 2 (AST)		Group 3 (ISO)		Group 4 (ISO+AST)	
	Control Col 1	$+Ca^{2+}$ Col 2	Control Col 3	$+Ca^{2+}$ Col 4	Control Col 5	$+Ca^{2+}$ Col 6	Control Col 7	$+Ca^{2+}$ Col 8
Mean 1	0.9427	1.0695	1.0331	0.9353	0.4584	0.2197	0.5237	0.3567
Mean 2	1.0417	1.002	1.0815	1.0968	0.4393	0.275	0.5693	0.457
Mean 3	1.0157	1.0215	0.9968	0.964	0.5029	0.2735	0.5758	0.3999

d **Sub c**

	Group 1 (w/o adds)		Group 2 (AST)		Group 3 (ISO)		Group 4 (ISO+AST)	
	Control Col 1	$+Ca^{2+}$ Col 2	Control Col 3	$+Ca^{2+}$ Col 4	Control Col 5	$+Ca^{2+}$ Col 6	Control Col 7	$+Ca^{2+}$ Col 8
Mean 1	1.0299	0.8208	0.9729	0.9999	0.7376	0.8114	2.5417	2.5418
Mean 2	1.0097	0.9776	0.9851	1.0478	0.7182	0.6759	2.5727	2.3531
Mean 3	0.9604	0.8271	1.0413	0.9984	0.8017	0.7677	2.6963	2.4537

CNPase

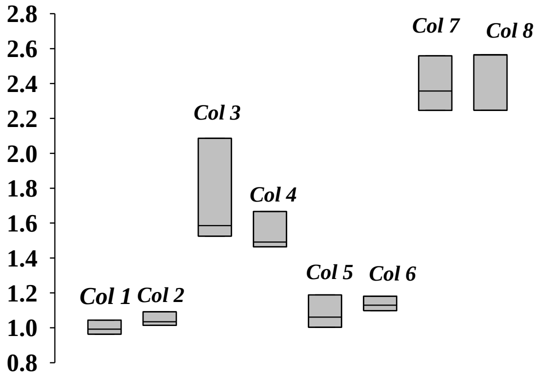

Sub b

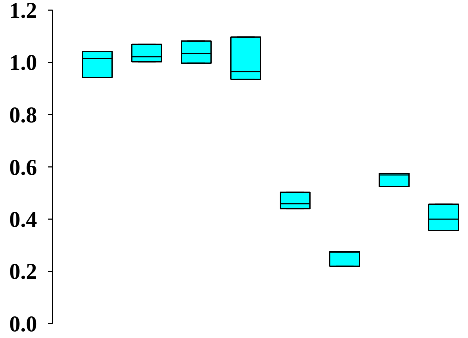

Sub c

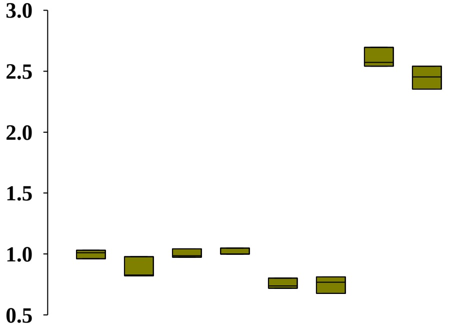

One Way Analysis of Variance All Pairwise Multiple Comparison Procedures (Student-Newman-Keuls Method) :

Comparison	Diff of Means	P<0.050
Col 3 vs. Col 1	0.732	Yes
Col 5 vs. Col 1	0.084	No
Col 7 vs. Col 1	1.387	Yes
Col 7 vs. Col 5	1.303	Yes
Col 4 vs. Col 2	0.494	Yes
Col 6 vs. Col 2	0.0897	No
Col 8 vs. Col 2	1.412	Yes
Col 8 vs. Col 6	1.322	Yes

Comparison	Diff of Means	P<0.050
Col 3 vs. Col 1	0.0371	No
Col 5 vs. Col 1	0.533	Yes
Col 7 vs. Col 1	0.444	Yes
Col 7 vs. Col 5	0.0893	Yes
Col 4 vs. Col 2	0.0323	No
Col 6 vs. Col 2	0.775	Yes
Col 8 vs. Col 2	0.626	Yes
Col 8 vs. Col 6	0.148	Yes

Comparison	Diff of Means	P<0.050
Col 3 vs. Col 1	0.000275	No
Col 5 vs. Col 1	0.248	Yes
Col 7 vs. Col 1	1.604	Yes
Col 7 vs. Col 5	1.851	Yes
Col 4 vs. Col 2	0.14	No
Col 6 vs. Col 2	0.124	Yes
Col 8 vs. Col 2	1.574	Yes
Col 8 vs. Col 6	1.698	Yes
